# Features of Aligners and Artificial Intelligence in Surgical–Orthodontic Protocol: A Narrative Communication

**DOI:** 10.3390/jcm15166433

**Published:** 2026-08-20

**Authors:** Andrea Varazzani, Louis Brochet, Alice Prevost, Nicolas Graillon, Pierre Bouletreau

**Affiliations:** 1 Maxillo-Facial Surgery, Facial Plastic Surgery, Stomatology and Oral Surgery Department, Lyon-Sud Hospital, Hospices Civils de Lyon, 69495 Pierre-Bénite, France; louis.brochet@chu-lyon.fr (L.B.); pierre.bouletreau@chu-lyon.fr (P.B.); 2 Plastic and Maxillo-Facial Surgery Department, University Hospital Center of Toulouse, 31059 Toulouse, France; prevost.a@chu-toulouse.fr; 3 Oral and Maxillofacial Surgery Department, CHU Conception, APHM, 13005 Marseille, France; nicolas.graillon@ap-hm.fr; 4 LBA UMR_T24, IFSTTAR, Aix-Marseille Université, 13015 Marseille, France

**Keywords:** surgical–orthodontic protocol, aligners, artificial intelligence, orthognathic surgery

## Abstract

**Background and Objectives:** Dentofacial deformities require a multidisciplinary surgical–orthodontic protocol (SOP) in which orthodontic preparation, orthognathic surgery, postoperative finishing, and retention are closely coordinated to achieve stable functional and aesthetic outcomes. The increasing adoption of clear aligners, digital workflows, and artificial intelligence (AI) has profoundly modified this therapeutic pathway. This communication examines the role of clear-aligner therapy and AI throughout the contemporary surgical–orthodontic protocol, with particular emphasis on their integration into clinical practice. **Discussion:** From the surgical perspective, clear aligners provide treatment outcomes comparable to those achieved with conventional fixed appliances while offering greater predictability of treatment duration through digital treatment planning. However, their use requires careful management of specific clinical aspects, including surgical timing, intraoperative anchorage, postoperative dentoalveolar stabilisation, transverse dimension management, and retention, all of which demand close collaboration between the orthodontist and the maxillofacial surgeon. The communication also summarises current AI applications in orthodontics and orthognathic surgery, including automated cephalometric analysis, image segmentation, treatment-decision support, virtual patient construction, clear-aligner setup, soft-tissue prediction, refinement-risk assessment, and remote monitoring. Although AI has significantly improved the efficiency, reproducibility, and standardisation of diagnosis and treatment planning, current evidence supports its use as a clinician-supervised decision-support technology rather than as an autonomous system capable of managing the entire orthodontic–surgical pathway. **Conclusions:** The integration of clear aligners, digital planning, and AI represents an important step toward a more personalised and efficient workflow, while continued clinical validation and multidisciplinary expertise remain essential for achieving predictable long-term outcomes.

## 1. Introduction

Dentofacial deformities are anomalies in the development and position of the jaws in relation to each other or to the skull. These bony bases support the dental arches. Their malposition leads to a number of functional impairments affecting mastication, phonation, breathing, and the temporomandibular joints. However, the aesthetic impact of these deformities cannot be overlooked, as it is often the main reason why patients undergo a surgical–orthodontic protocol [1].

These anomalies are characterised according to the affected bony base(s) (mandible and/or maxilla), the nature of the deformity (excess or deficiency), and the spatial dimension involved (vertical, sagittal, and/or transverse).

The clinical and morphological presentation often results from the combination of several anomalies. A dentofacial deformity usually includes abnormalities in all three spatial dimensions, although the classification generally refers to the developmental problem that predominates over the coexisting anomalies. These disorders are commonly grouped into major syndromes, including long face, short face, dento-skeletal Class II or III, and facial asymmetry.

Orthognathic surgery (from the Greek ortho, meaning “to straighten,” and gnathos, meaning “jaw”) involves the surgical repositioning of the jaws and aims to correct dentofacial deformities in order to improve function, aesthetics, and psychosocial well-being. To define the treatment objectives, criteria of normality regarding facial harmony, dental occlusion, and the position of the cranio-maxillofacial skeletal bases must be considered [2]. These criteria define a face that is aesthetically and structurally balanced while ensuring optimal orofacial function. Dental occlusion is considered optimal when it corresponds to Class I occlusion, the maxillary and mandibular dental midlines are aligned, and the maxillary arch is wider than the mandibular arch, overlapping it by approximately 2 mm. Various occlusal disorders may therefore be associated with dentofacial deformities, including dental Class II or III malocclusion, deep bite, open bite, crossbite, and others.

Dental malocclusions caused by abnormalities in the position of the skeletal bases cannot be corrected predictably or stably by orthodontic treatment alone. The extent of the required dental movements exceeds the limits of dentoalveolar compensation and exposes the patient to a high risk of relapse. Only surgical repositioning of the skeletal bases allows the orthodontist to complete the treatment successfully. The maxillofacial surgeon performs various maxillary and/or mandibular osteotomies to correctly reposition the dental arches. The management of dentofacial deformities therefore usually requires the implementation of a surgical–orthodontic protocol (SOP). Developmental abnormalities of the skeletal bases induce and maintain dental compensations that must first be eliminated to allow correct skeletal repositioning.

An SOP consists of four consecutive phases.

(1) Preoperative orthodontic preparation phase: This phase may last from 6 to 18 months [3]. Orthodontic preparation may be performed using clear aligners or with conventional fixed appliances, either vestibular or lingual.
(2) Surgical phase: The most commonly performed maxillary osteotomy worldwide is the Le Fort I osteotomy. The maxilla may also be segmented to increase its transverse dimension. The most frequently performed mandibular osteotomy is the bilateral sagittal split osteotomy (BSSO), so called because the osteotomy follows a sagittal plane. Other mandibular osteotomies (such as the inverted-L osteotomy, vertical ramus osteotomy, and short osteotomy) and alternative maxillary osteotomies have also been described, although they are used less frequently [4].
(3) Orthodontic finishing phase: It usually begins approximately one month after surgery, lasts on average six months, and ends when a balanced occlusion has been achieved in all three planes of space.
(4) Retention phase: The most common retainers are bonded lingual wires attached to the incisors and removable thermoplastic retainers. The optimal duration of retention remains debated but, depending on the periodontal condition, lifelong retention may be recommended.

The SOP can be modified in selected cases by eliminating the orthodontic preparation phase (surgery-first approach) or shortening it (early surgery approach). These alternative protocols require even closer collaboration between the surgeon and the orthodontist [5,6,7].

The introduction of intraoral scanners has profoundly changed the role of digital imaging in the surgical–orthodontic protocol. The SOP is now supported by three-dimensional digital treatment-planning software, including virtual setup platforms (ClinCheck, Insignia, OrthoAnalyzer, etc.), many of which incorporate machine-learning algorithms. These technologies have markedly reduced the need for plaster models and conventional lateral cephalograms. In the surgical phase, virtual surgical planning (VSP) has become routine in most maxillofacial surgery departments, allowing a more comprehensive analysis of dentofacial deformities. These digital tools also improve communication among the surgeon, the orthodontist, and, most importantly, the patient [8].

Artificial intelligence is increasingly being used in orthodontics, clear-aligner therapy, and orthognathic surgery. However, the available literature mainly describes separate applications in these fields, whereas evidence supporting a single, fully integrated AI-based protocol combining clear aligners and orthognathic surgery remains limited [9,10,11,12].

At present, AI should therefore be considered a collection of complementary tools that support different phases of treatment rather than an autonomous system capable of managing the entire orthodontic–surgical pathway.

## 2. Discussion: Clear Aligners

From the maxillofacial surgeon’s perspective, orthodontic preparation with aligners offers no particular disadvantages or advantages compared with conventional preparation using either vestibular or lingual fixed appliances. The criteria for operability in patients with maxillomandibular dysmorphia must likewise be fulfilled at the end of orthodontic preparation to achieve a stable surgical occlusion: interarch coordination must allow for canine and molar Class I relationships, physiological anterior overbite, aligned dental midlines, and physiological intercanine and posterior transverse dimensions. However, orthodontic preparation with aligners has several specific features when used as part of a surgical–orthodontic protocol.

### 2.1. Timing of Surgery

Apart from a surgery-first protocol, which is perfectly feasible with aligners, a conventional surgical–orthodontic protocol consists of four distinct phases, as described above, and orthognathic surgery can only be performed after completion of the orthodontic preparation.

In cases treated with conventional orthodontic brackets and wires, the duration of the orthodontic preparation phase is unpredictable but typically ranges from 9 to 18 months [13]. This frequently prolongs the overall treatment time because the surgeon may not be available when the patient is ready for surgery, especially in the current context of limited surgical operating time [14].

Thanks to treatment simulation software, aligner therapy offers the advantage of accurately predicting the completion date of orthodontic preparation, thereby allowing optimisation of surgical scheduling for both the patient and the treating clinicians [15]. With conventional fixed appliances, greater clinical experience is required to accurately predict the end of treatment [16].

### 2.2. Anchorage

During surgery and throughout the early postoperative period (the first four weeks), it is necessary to stabilise the maxillary and mandibular arches using wires (during surgery) or elastics (during the postoperative guidance phase). In conventional vestibular treatment, metal wires or elastics are attached to surgical hooks placed by the orthodontist before surgery. In patients treated with aligners, the orthodontist must bond metal vestibular posts a few days before surgery to provide anchorage for intraoperative wires and postoperative elastics.

The quality of bonding of these posts is important because, in addition to the discomfort and surgical instability caused by the loss of several posts, there is a risk that one or more posts may become dislodged into the osteotomy sites during surgery, potentially leading to postoperative infectious complications. Furthermore, some posts are manufactured from transparent materials to improve aesthetics. However, these materials are often radiolucent and therefore difficult or impossible to detect on radiographic imaging. Consequently, if a post is inadvertently lost during surgery, intraoperative radiographs may not be useful in locating it.

### 2.3. Postoperative Dentoalveolar Stability

Patients usually see their orthodontist approximately two weeks after orthognathic surgery. It is therefore essential that the dental alignment and levelling achieved during the presurgical phase remain stable throughout the immediate postoperative period. In conventional orthodontic treatment, the archwire and brackets provide excellent dentoalveolar stabilisation. In patients treated with aligners, however, the immediate postoperative aligner planned by the orthodontist must be inserted as soon as possible because it alone provides dentoalveolar support. Ideally, the surgeon should place the aligner at the end of the procedure. This postoperative aligner must be relieved at the sites of the vestibular posts to avoid interference during insertion.

### 2.4. Posterior Transverse Dimension

Correction of the posterior transverse dimension is fundamental to the stability of orthodontic treatment. When this skeletal dimension is surgically modified (posterior expansion), it is essential that the new maxillary arch morphology has already been incorporated into the postoperative aligner series.

Orthodontic preparation with aligners therefore requires closer collaboration between the orthodontist and the surgeon. The surgeon must inform the orthodontist of the planned amount of posterior transverse correction included in the surgical setup. This information must be provided sufficiently in advance to allow the orthodontist enough time to modify the postoperative aligner series.

Current digital workflow tools facilitate this exchange of information in several ways. For example, the maxillofacial surgeon can obtain an optical scan of the expanded maxillary arch according to the planned surgical setup and send the resulting STL file to the orthodontist, thereby accelerating the fabrication of the postoperative aligners. However, this exchange of information must be anticipated to ensure that the patient receives the postoperative aligners before the date of surgery.

An alternative—and probably more logical—approach is for the orthodontist to define the ideal posterior transverse correction. The STL file of the maxillary arch with the desired transverse expansion can then be transmitted to the surgeon, who performs the virtual surgical setup, including the fabrication of the surgical splints, according to the maxillary arch form planned by the orthodontist. This approach also provides the orthodontist with sufficient time to deliver the modified aligners before surgery.

### 2.5. Retention of the Posterior Transverse Dimension

It is well recognised that surgical correction of the posterior maxillary transverse dimension (posterior maxillary expansion) has a significant tendency to relapse. Proffit et al. considered it one of the least stable procedures in orthognathic surgery [17]. It is therefore essential to maintain the corrected posterior transverse dimension following any maxillary expansion greater than 2 mm.

In patients treated with conventional fixed appliances, retention is usually achieved with a transpalatal arch attached to bands fitted with palatal sheaths on the maxillary first molars. In patients treated with aligners, retention can be achieved using several different approaches [18]. Although the orthodontist may place metal bands with palatal sheaths before surgery, many patients who choose aligner therapy for its aesthetic advantages are reluctant to accept this option. In such cases, a palatal splint or a bone-supported distractor may be used to maintain the posterior transverse dimension. Again, the postoperative aligner must be relieved on its palatal aspect to avoid interference.

## 3. Discussion on Artificial Intelligence

Normally, surgical–orthodontic planning requires the collaboration of at least three figures: the orthodontist, maxillofacial surgeon and patient. So, AI should acquire the skills and understand the needs of these three figures in order to be able to create an SOP independently.

As far as the ability to learn is concerned, there is no doubt that a machine has much greater capabilities than a human being in acquiring and organising information, especially considering that the amount of information for each patient has grown so much that it has become impossible to fully comprehend.

The result of bone movements at the soft-tissue level are very difficult to predict, especially in the labial and mental region, as the skin, subcutaneous fat tissue, muscles and mucosa have different elasticity coefficients, although the prediction has improved significantly recently. Furthermore, these parameters are different in the same patient depending on age and clearly differ from one patient to another, depending on ethnicity, gender, etc.

Another difficulty that AI still has is in understanding the ambiguous needs that patients have about their face, what they want to change and most of all what they want to preserve of their face. This is probably the hardest point to solve because normally machines struggle with ambiguous data.

### 3.1. Categories of Algorithms

During SOP the most widely used algorithms are deep-learning computer-vision models, particularly convolutional neural networks and segmentation architectures such as U-Net. These models analyse cephalometric radiographs, CBCT volumes, facial images and digital dental models. Their main applications include automatic landmark detection, segmentation of skeletal and dental structures, classification of skeletal malocclusions, assessment of facial asymmetry and prediction of postoperative soft-tissue changes [19,20].

Classical supervised machine-learning models are also frequently used when the available data consist of numerical clinical or cephalometric variables. These algorithms include logistic regression, support-vector machines, random forests, decision trees and gradient-boosting methods such as XGBoost. Their purpose is generally to classify patients into treatment categories, for example, orthodontic camouflage versus orthognathic surgery, or to estimate the probability of a specific event such as the need for clear-aligner refinement [21,22].

Artificial neural networks and multilayer perceptrons are used for similar classification and prediction tasks. They may analyse structured measurements alone or combine them with image features extracted by convolutional networks.

Other emerging approaches include regression models for predicting continuous outcomes, such as postoperative landmark positions, facial soft-tissue displacement or treatment duration. Generative models, biomechanical surrogate models and patient-specific digital-twin concepts are also being investigated, particularly for predicting facial changes after skeletal repositioning. However, these applications remain mostly experimental and require further clinical validation.

Finally, computer-vision algorithms are used in remote aligner monitoring. These systems analyse smartphone photographs to assess aligner seating, treatment progression and possible tracking problems. Some platforms produce a simple “GO” or “NO-GO” recommendation indicating whether the patient should proceed to the next aligner [23,24].

### 3.2. Inputs and Outputs

The inputs during SOP depend on the specific clinical task. For diagnostic and cephalometric applications, AI systems may analyse lateral or posteroanterior cephalograms, CBCT datasets, facial photographs and clinical variables. Their outputs include anatomical landmark coordinates, cephalometric measurements, skeletal classifications and asymmetry indices.

When AI is used to support the decision between orthodontic treatment alone and orthognathic surgery, the inputs usually consist of hard- and soft-tissue cephalometric variables, such as SNA, SNB, ANB, Wits appraisal, overjet, overbite, incisor inclination and facial-profile measurements. The output is generally a binary classification or a probability indicating whether the patient is more likely to require orthodontic camouflage or surgical correction.

For three-dimensional treatment planning, the system may receive CBCT or CT images, intraoral scans, facial scans and bite registrations. The outputs can include segmented maxillary and mandibular structures, digital crowns and roots, alveolar-bone models and a combined virtual patient.

In clear-aligner planning, the inputs include the initial tooth positions, target occlusion, planned tooth movements, attachments, interproximal reduction and clinician-defined movement constraints. The output is a virtual setup composed of sequential tooth positions, the number of aligners, attachment prescriptions and the amount of interproximal reduction required.

In orthognathic planning, AI may also analyse planned maxillary, mandibular and chin movements. The outputs can include repositioned skeletal segments, virtual intermediate and final occlusions, predicted postoperative facial surfaces and displacement maps.

For aligner-refinement prediction, variables such as compliance, interproximal reduction and tooth-specific movements can be used to estimate whether additional refinement aligners will be needed. Wolf et al. used regularised logistic regression and XGBoost for this purpose and identified specific movements, such as mandibular incisor rotation, as being associated with a higher risk of refinement.

### 3.3. Integration into Clinical Workflows

AI is generally integrated into existing orthodontic and surgical software as a semi-automated decision-support tool.

During the diagnostic phase, radiographs and CBCT datasets are imported into dedicated software. AI automatically identifies landmarks, segments anatomical structures and calculates measurements. The orthodontist or surgeon then verifies the results and corrects possible errors before using them for diagnosis and treatment planning.

A complete digital patient may be created by combining CBCT skeletal data, intraoral scans, facial scans and photographs. AI can assist with segmentation and registration, producing a common three-dimensional model that can be transferred to orthodontic setup and virtual surgical-planning software.

The orthodontic and surgical plans must then be coordinated. The orthodontist develops the dental setup, while the surgeon defines the skeletal correction. These two simulations are combined to ensure that the planned tooth movements are compatible with the surgical repositioning and that the final occlusion is both achievable and stable.

Following approval, the orthodontic plan is transferred to the clear-aligner platform, where tooth movements are staged and aligners are manufactured. Commercial systems may use proprietary AI or optimisation algorithms during this process, but their precise architecture and training data are rarely disclosed in the scientific literature.

The surgical plan can be transferred to the operating room using occlusal splints, cutting guides, patient-specific fixation devices or navigation systems. AI supports the planning process through segmentation, landmark detection and outcome prediction.

After surgery, a new intraoral scan may be obtained to compare the actual result with the original virtual setup. New finishing aligners can then be created to correct residual discrepancies. Remote-monitoring systems may subsequently evaluate serial intraoral photographs and alert the clinician in cases of poor tracking or inadequate aligner fit.

### 3.4. Clinical Interpretation

The most established applications of AI in an aligner-based orthodontic–orthognathic pathway are automated cephalometric analysis, CBCT segmentation, treatment-decision support, clear-aligner setup, soft-tissue prediction, refinement-risk assessment and remote monitoring.

Nevertheless, the current evidence concerns individual tasks rather than a single integrated system. Clear aligners can be used successfully in selected orthognathic patients, including surgery-first protocols, but the available studies remain heterogeneous and are often based on retrospective cohorts, case series or case reports [25].

## 4. Conclusions

Orthodontic preparation with aligners does not represent an obstacle within a surgical–orthodontic protocol [26]. It may even reduce the overall treatment time by optimising surgical scheduling. However, this orthodontic technique requires excellent collaboration between the orthodontist and the maxillofacial surgeon [27]. In addition, clinicians need to be familiar with the specific features of aligner-based preparation.

AI should currently be viewed as a collection of clinician-supervised tools embedded at different stages of the orthodontic–surgical workflow. It can improve efficiency, standardisation, and treatment visualisation, but it does not replace the multidisciplinary judgement of the orthodontist and the maxillofacial surgeon.

## Data Availability

All supplementary information regarding the sources can be obtained by contacting the corresponding author.
